# The risk factors for urinary incontinence in female adults with chronic cough

**DOI:** 10.1186/s12890-022-02069-w

**Published:** 2022-07-18

**Authors:** Cunzhen Yang, Zien Feng, Zhiyin Chen, Dongting Xu, Yuling Li, Kefang Lai, Fang Yi

**Affiliations:** State Key Laboratory of Respiratory Disease, National Clinical Research Center for Respiratory Disease, National Center for Respiratory Medicine, Guangzhou Institute of Respiratory Health, The First Affiliated Hospital of Guangzhou Medical University, 28 middle Qiaozhong Rd, Liwan District, Guangzhou, Guangdong People’s Republic of China

**Keywords:** Urinary incontinence, Female, Chronic cough, Risk factors

## Abstract

**Background:**

Female patients with chronic cough are more likely to suffer from urinary incontinence (UI). However, there are few data in regard of risks related with UI in female adults with chronic cough.

**Method:**

We recruited female adult patients with chronic cough from the cough specialist clinic. Demographic information and clinical characteristics including age, BMI, duration of cough, severity of cough, nature and timing of cough, cough triggers, concomitant symptoms, comorbidities and UI condition were collected. The demographics and clinical features of patients with UI and those without UI were compared.

**Result:**

A total of 700 female patients with the main symptom of chronic cough were included, of whom 351 (50.1%) presented with UI. As compared with patients without UI, patients with UI showed a longer mean age (years) (49.5 vs. 42.4, *p* < 0.001), a more severe cough symptom (median of cough Visual Analogue Scale: 65 vs. 50, *p* < 0.001), a higher prevalence of chronic sinusitis (17.6% vs. 8.6%, *p* = 0.002), and combined with a higher incidence of abdominal muscle pain due to cough (39.6% vs. 18.7%, *p* < 0.001).In addition, patients in UI group whose cough were more easily triggered by exercise (28.2% vs. 17.2%, *p* = 0.048). Multivariate logistic regression analysis indicated the above five variables were risk factors for UI in female adult patients with chronic cough.

**Conclusion:**

Urinary incontinence is a common complication in female patients with chronic cough. Older age, severe cough, combing with a higher proportion of chronic sinusitis and abdominal muscle pain, a cough easily triggered by exercise are identified as risk factors for urinary incontinence. We should pay more attention to female chronic coughers with these risk factors in clinics.

**Supplementary Information:**

The online version contains supplementary material available at 10.1186/s12890-022-02069-w.

## Introduction

Chronic cough is defined as cough presenting as the sole or predominant symptom and lasting for more than 8 weeks, with no obvious abnormalities in chest imaging. Chronic cough accounts for up to 10 to 38% patients [1] in respiratory clinics, and cough variant asthma (CVA), eosinophilic bronchitis (EB), gastroesophageal reflux cough (GERC), upper airway cough syndrome (UACS), and atopic cough (AC) are the common causes of chronic cough. In western special cough clinics, patients with chronic cough were middle-aged and elderly women predominant [2–4]. However, there was no significant difference on the prevalence of chronic cough between males and females in China both in the cough clinics and the respiratory clinics [5–7]. Female patients usually complain about more severe cough [5] and are more likely to combine with comorbidities such as abdominal muscle pain and urinary incontinence (UI), which affect patients’ quality of life and increase their psychological burden greatly. Urinary incontinence refers to the involuntary flow of urine from the urethra.

The incidence of UI in female patients with chronic cough is much higher than that in males, a shorter urethra and weaker sphincter tension presented in females should be the main results. Meanwhile, the presence of UI is also related to the pelvic floor dysfunction provoked by pregnancy, vaginal delivery and obstetrical and gynecological surgery, all of which are female-specific [8–10]. The prevalence of UI in females with chronic cough was inconsistent among previous reports, but it is usually more than 30% [11–16], and can even be up to be 50% [12, 16]. Previous studies showed that older age, high BMI (Body mass index, kg/m^2^), history of vaginal delivery, obstetrical and gynecological surgery and chronic cough are common risk factors of UI in adult women [13, 17–20, 25–29]. However, it is unknown which clinical characteristics are risk factors for UI in patients with chronic cough. Therefore, the aim of this study is to investigate the risk factors of UI in adult female patients with chronic cough by comparing the demographic and clinical characteristics of patients with UI and those without UI.

## Method

### Subjects

Patients with cough who visited the specialist cough clinics of the First Affiliated Hospital of Guangzhou Medical University from January 2006 to December 2018 were asked to complete a standard case report form (CRF). An additional file shows the CRF in more detail (see Additional file 1). Coughers met the inclusion criterion as following were enrolled in this study: (1) Female gender, (2) aged more than 18 years, (3) cough as the only or predominant symptom lasting more than 8 weeks, (4) chest imaging showed no obvious abnormalities. Exclusion criterion: (1) information about urinary incontinence was not recorded, (2) age or duration of cough was not recorded. Basing on the above criterion, patients with UI were regarded as cases, while those without UI were deemed as controls.

### Study design

This was a retrospective observational study. Investigators assisted patients in filling in a CRF the first time when patients came to the clinics. Demographics and cough-related clinical characteristics were included in the CRF, which detailly contained age, BMI, duration of cough, timing of cough, dry cough or productive cough, severity of cough, cough triggers, concomitant symptoms, comorbidities and previous treatment for cough.

Daytime/Night-time Cough symptom score (CSS) and cough Visual Analogue Scale (VAS) were used to evaluate the frequency and the severity of cough. Daytime/Night-time CSS are ranging from 0 to 5, 0 for no cough, 5 for uncontrollable cough, and inability to perform daily activities or fall asleep [21]. The cough VAS is a scale ranging from 0 to 100. Zero represents no cough and 100 represents the most severe cough.

Etiological diagnostic workflow and criteria are based on cough guidelines by the American College of Chest Physicians and the Chinese Medical Association [22–24]. We classified patients according to etiology into asthmatic cough (including cough variant asthma and classic asthma with cough as the main symptom), EB, GERC, UACS, AC, as well as rare and uncommon etiologies. In addition to the categories mentioned above, we grouped patients together with unexplained chronic cough (UC) and chronic refractory cough (CRC) with diagnosable etiology and failed in repeatedly treatment. The study was approved by the Ethics Committee of the First Affiliated Hospital of Guangzhou Medical University (Medical Research 2,020,150). Neither this study involved any therapeutic interventions, nor did it impose any additional burden.

### Statistical analysis

Data were presented as percentages(frequencies), means (standard deviations, SD), or medians (interquartile range, IQR). Correlation tests were performed between variables to identify those with collinearity (r > 0.7). Pearson correlation coefficients were used to reflect correlations between continuous variables, Spearman and Kendall's tau-b correlation coefficients were used to reflect correlations between categorical variables. Two independent samples t-test was used for normally distributed data, Mann–Whitney U test for non-normally distributed data, χ2 test or Fisher exact test for categorical data, and Bonferroni correction test for multiple comparisons. BMI (kg/m^2^) was classified into four grades: underweight, < 18.5; normal, 18.5–23.9; overweight, 24–27.9; obesity, ≥ 28. Age was categorized into five grades: I, 18–30 years; II, 31–40 years; III, 41–50 years; IV, 51–60 years; V, > 60 years. Variables associated with UI from univariate analysis (*P* < 0.1) were included in multivariate analysis. The final independent risk factor of UI was determined by the stepwise logistic regression analysis. Based on the age grade, we set the dummy variable for "age" in the logistic regression analysis. *P* < 0.05 was considered as a statistically significant difference. The software used for data analysis was SPSS 25.0 (IBM Corporation, Armonk, NY) and GraphPad Prism 8.

## Results

### Demographics and clinical characteristics

**Fig. 1:**
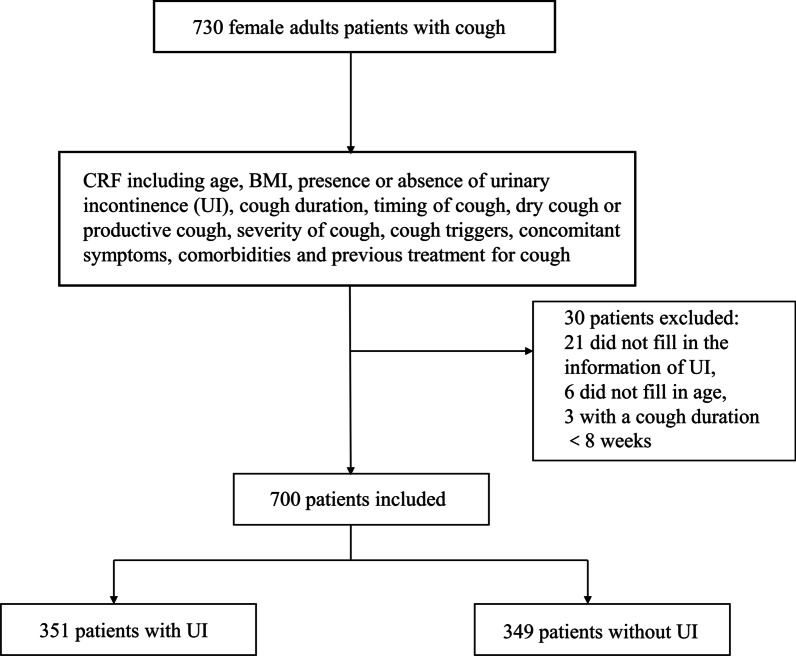
Flow chart. CRF, Case report form; UI, Urinary incontinence

A total of 730 adult female patients were recruited from January 2006 to December 2018. Among whom, 30 cases were excluded, including 21 cases who did not fill in the presence or absence of UI, 6 cases who did not fill in their age, and 3 cases with a cough duration less than 8 weeks. The remaining 700 patients with a mean age of 45.9 years old and a median cough duration of 24 months were finally enrolled into the analysis. Among these 700 patients, 351 patients had reported UI (50.1%). The flow chart was shown in Fig. 1.

The mean age and the median cough duration of patients with UI were significantly higher than that of patients without UI (49.5 ± 13.33 vs. 42.4 ± 13.85, *p* < 0.01; 36(12–108) vs. 18(6–48), *p* < 0.01 respectively). The patients with UI showed higher daytime CSS, night-time CSS, cough VAS score and lower proportion of single cough as compared with patients without UI (Table 1). In addition, patients with UI were more likely to be sensitive to “dust”, “cooking fume”, “cold air”, “supine position”, “cigarette smoke”, “exercise” as cough triggers and had significantly higher proportion of chest tightness, upper abdominal pain, chronic sinusitis and hypertension than those without UI (Table 1).

**Table 1:** Baseline demographic and clinical characteristics

	Total (n = 700)	Urinary incontinence (n = 351)	Without urinary incontinence (n = 349)	*p-value*
Age(years)	45.94 ± 14.01	49.46 ± 13.33	42.39 ± 13.85	< 0.001*
Cough duration (months)	24(8–84)	36(12–108)	18(6–48)	< 0.001*
BMI (kg/m^2^)	22.6 ± 3.3(n = 370)	23.2 ± 3.5(n = 193)	21.9 ± 3.0(n = 177)	< 0.001*
Daytime CSS	3(2–4)	3(2–4)	3(2–3)	0.001*
Nighttime CSS	2(1–2)	2(1–3)	2(1–2)	0.001^*^
Cough VAS	60(50–80)	65(50–80)	50(40–70)	< 0.001^*^
Dry cough, %	52.2(360/690)	51.7(180/349)	52.6(180/342)	0.811
A single cough, %	25.6(156/609)	21.0(66/315)	30.6(90/294)	0.006^*^
Continuous cough, %	69.7(435/624)	70.4(226/321)	69(209/303)	0.698
Seasonal, %	29.8(200/671)	31.6(108/342)	28.0(92/329)	0.306
Timing of cough				
Daytime, %	85.0(590/694)	87.6(305/348)	82.4(285/346)	0.052
Before sleep, %	60.8(421/692)	65.7(228/347)	55.9(193/345)	0.009^*^
Night, %	52.6(364/692)	57.6(200/347)	47.5(164/345)	0.008^*^
Morning, %	46.4(321/692)	49.3(171/347)	43.5(150/345)	0.126
Triggers				
Dust, %	51.2(354/691)	57.3(199/347)	45.1(155/344)	0.001^*^
Cooking fume, %	56.4(390/692)	62.8(218/347)	49.9(172/345)	0.001^*^
Cold air, %	54.3(375/691)	58.7(203/346)	49.9(172/345)	0.020^*^
Common cold, %	59.3(412/693)	62.2(216/347)	56.6(196/346)	0.133
Supine position, %	23.6(163/690)	29.4(102/347)	17.8(61/343)	< 0.001^*^
Cigarette smoke, %	55.3(383/693)	63.2(220/348)	47.2(163/345)	< 0.001^*^
Exercise, %	20.2(139/689)	23.2(80/345)	17.2(59/344)	0.048^*^
Talking, %	37.6(260/692)	39.9(138/346)	35.3(122/346)	0.209
Alcohol, %	2.9(17/594)	2.9(9/306)	2.8(8/288)	0.905
History of medication				
Oral corticosteroids, %	19.9(112/563)	22.8(66/289)	16.8(46/274)	0.072
Inhaled corticosteroids, %	34.6(197/570)	37.5(110/293)	31.4(87/277)	0.124
Antitussive drugs, %	74.6(447/599)	75.8(229/302)	73.4(218/297)	0.495
Concomitant symptoms				
Sneezing, %	36.7(256/697)	38.6(135/350)	34.9(121/347)	0.311
Rhinocnesmus, %	25.8(179/694)	28.0(91/347)	23.6(82/347)	0.193
Nasal congestion, %	28.1(195/693)	28.7(100/348)	27.5(95/345)	0.726
Postnasal dripping, %	23.6(164/694)	25.3(88/348)	22.0(76/346)	0.303
Chest tightness, %	31.2(216/693)	35.0(122/349)	27.3(94/344)	0.030^*^
Shortness of breath, %	22.8(158/693)	25.9(90/348)	19.7(68/345)	0.054
Acid regurgitation, %	19.3(134/696)	20.9(73/350)	17.6(61/346)	0.280
Nausea, %	20.4(142/696)	22.0(77/350)	18.8(65/346)	0.293
Belching, %	21.4(149/695)	22.6(79/349)	20.2(70/346)	0.440
Heartburn, %	8.2(57/694)	10.0(35/349)	6.4(22/345)	0.080
Upper abdominal pain, %	8.8(61/696)	11.4(40/350)	6.1(21/346)	0.012^*^
Throat clearing, %	34.9(242/693)	38.2(133/348)	31.6(109/345)	0.067
Itchy throat, %	60.4(420/695)	64.3(225/350)	56.5(195/345)	0.036^*^
Itching below the throat, %	29.9(209/698)	28.3(99/350)	31.6(110/348)	0.338
Pharyngeal foreign body sensation, %	34.1(221/649)	38.2(128/335)	29.6(93/314)	0.021^*^
Abdominal muscle pain due to cough, %	29.2(204/699)	39.6(139/351)	18.7(65/348)	< 0.001^*^
Comorbidities				
Gastrointestinal disorders, %	24.4(164/672)	24.7(83/336)	24.2(81/336)	0.857
Hypertension, %	14.9(100/670)	19.5(65/334)	10.4(35/336)	0.001^*^
Allergic rhinitis, %	25.3(170/672)	22.6(76/336)	28.0(94/336)	0.110
Chronic sinusitis, %	12.6(85/672)	17.6(59/336)	8.6(29/336)	0.002^*^

Data presented as mean ± SD or median (IQR) or percentage (positive cases/ total cases in groups except for missing value). Patients with UI vs patients without UI

*p < 0.05

*UI* Urinary incontinence, *CSS* Cough symptom score, *VAS* Visual analogue scale, *OCS* Oral corticosteroids, *ICS* Inhaled corticosteroids

**Fig. 2:**
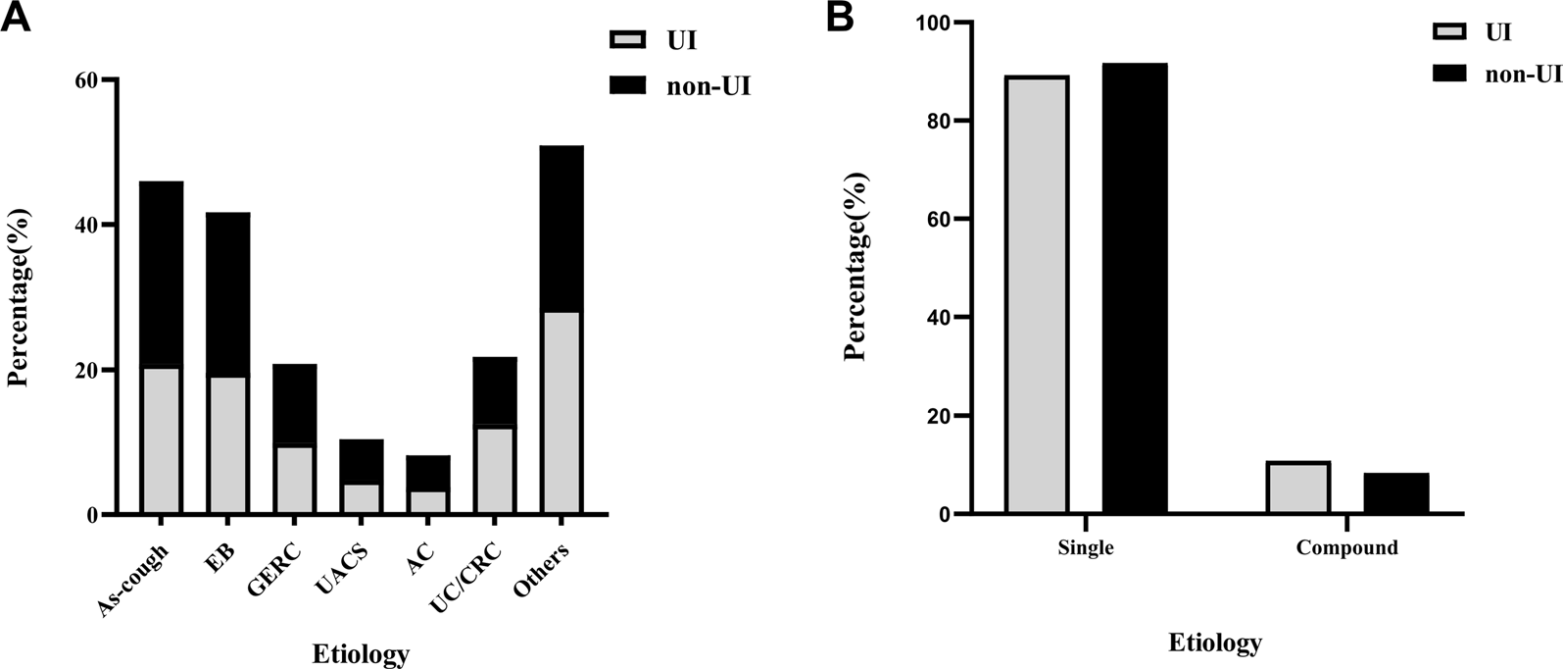
**A** Etiological distribution in patients with UI and patients without UI. **B** Proportion of single etiology and compound etiology between patients with and without UI. UI, Urinary incontinence; EB, Eosinophilic bronchitis; GERC, Gastroesophageal reflux cough; UACS, Upper airway cough syndrome; AC, Atopic cough; UC, Unexplained chronic cough; CRC, Chronic refractory cough

Of the 700 enrolled patients, 633 with a single cause, including asthmatic cough (146, 23.1%), EB (132, 20.9%), GERC (66, 10.4%), UACS (33, 5.2%), AC (26, 4.1%), other rare causes (161, 25.4%) and UC/CRC (69, 10.9%). There was no significant difference in etiological distribution between patients with and without UI (Fig. 2A), and no significant difference in the proportion of single etiology and compound etiology between the two groups (Fig. 2B).

Two demographic factors "age" and "BMI" were closely related to the occurrence of UI, thus we conducted a sub-group analysis for these two variables (Table 2). The incidence of UI increased with the increase of age and BMI. In addition, most of the patients with UI were middle-aged and elderly people (> 40 years old), while the patients without UI were younger (< 40 years old). The proportion of overweight and obesity in UI patients was 1.8 and 3.1 times higher than that in those with normal BMI.

**Table 2:** Relationship between urinary incontinence and age or BMI of different grades

	Occurrence rate% (95%*CI*)	Cases in group UI (%)	Cases in group without UI (%)	Univariate analysis	
				*OR*(95%*CI*)	*p value*
Age(years)	50.1 (46.4–53.9)	351 (50.1%)	349 (49.9%)		< 0.001
18–30	26.2 (17.7–34.6)	28 (8.0%)	79 (22.6%)	1	
31–40	42.0 (34.5–49.4)	73 (20.8%)	101 (28.9%)	2.039 (1.205–3.450)	0.008
41–50	59.1 (51.1–67.0)	88 (25.1%)	61 (17.5%)	4.070 (2.370–6.990)	< 0.001
51–60	57.5 (49.6–65.4)	88 (25.1%)	65 (18.6%)	3.820 (2.232–6.536)	< 0.001
> 60	63.2 (54.4–72.1)	74 (21.1%)	43 (12.3)	4.855 (2.741–8.602)	< 0.001
BMI (kg/m^2^)	52.2 (47.0–57.3)	193 (52.2%)	177 (47.8%)		0.008
Underweight (< 18.5)	39.4 (21.8–57.0)	13 (6.7%)	20 (11.3%)	0.710 (0.337–1.495)	0.367
Normal (18.5–23.9)	47.8 (41.3–54.3)	109 (56.5%)	119 (67.2%)	1	
Overweight (24–27.9)	62.8 (52.4–73.2)	54 (28.0%)	32 (18.1%)	1.842(1.108–3.064)	0.019
Obesity (≥ 28)	73.9 (54.5–93.3)	17 (8.8%)	6 (3.4%)	3.093 (1.177–8.130)	0.022

*CI*, Confidence interval; BMI, Body mass index; UI, Urinary incontinence

### The multivariate analysis of risk factors

The univariate analysis showed that there were obvious differences on 27 variables between groups (all *p* < 0.1), including age, duration of cough, BMI, daytime and night-time CSS, cough VAS, a single cough, cough at daytime, cough before sleep, cough at night, oral corticosteroids use, accompanied by chest tightness, shortness of breath, heartburn, throat clearing, upper abdominal pain (stomachache), itchy throat, foreign body sensation in the pharynx, combining with chronic sinusitis, hypertension, abdominal pain due to cough and cough triggers (e. g. dust, cooking fumes, cold air, supine position, cigarette smoke, and exercise) All the variables mentioned above were not collinear and could be entered in the logistic regression analysis. However, the variable of BMI was finally excluded considering there was so much missing data, which may cause a great impact on the results.

In multivariate analysis, older age, higher cough VAS (OR = 1.027), combined with chronic sinusitis (OR = 1.918), cough easily triggered by exercise (OR = 1.736), and abdominal muscle pain due to cough (OR = 3.167) were independent risk factors for UI in female patients with chronic cough. Compared with younger patients, patients over 40 years old are more likely to be complicated with UI, especially in patients with an age over 60 years old, whose risk of UI is about 5.5 times higher than that of younger people aged 18–30 years (OR = 5.471) (Table 3).

**Table 3:** Multivariate logistic regression analysis on risk factors of UI in female patients with chronic cough

	Multivariate analysis		
	*OR*	95% CI	*P value*
Age(years)		< 0.001	
I 18–30	1		
II 31–40	2.087	1.022–4.264	0.043
III 41–50	3.801	1.798–8.036	< 0.001
IV 51–60	2.981	1.440–6.171	0.003
V > 60	5.471	2.455–11.954	< 0.001
Cough VAS	1.027	1.016–1.038	< 0.001
Combining with chronic sinusitis	1.918	1.026–3.588	0.041
Cough triggered by exercise	1.736	1.036–2.907	0.036
Abdominal muscle pain due to cough	3.167	1.931–5.194	< 0.001

VAS, Visual analogue scale; *CI, Confidence interval*

Subsequently, 10 variables associated with UI including “age, duration of cough, cough VAS, Daytime CSS, Nighttime CSS, a single cough, combining with chronic sinusitis, concomitant with sneeze, cough triggered by exercise, abdominal muscle pain due to cough” were enrolled in the multivariate analysis to create another multivariate model. The result (See in additional file 2) was extremely similar to what shown in Table 3. Even though BMI was not included in our formal data analysis, two attempts were made about this variable: (1) enrolled “BMI” and all others variables with *p* < 0.1 in the univariate analyses into logistic regression; (2) enrolled “BMI” and the 10 variables mentioned above in the logistic regression. In neither attempt, “BMI” entered the regression equation (data was not shown).

## Discussion

This is the first study to investigate the risk factors of UI in female adult patients with chronic cough. In this study, we found that the incidence of UI in adult female chronic cough patients was 50.1%. In addition, older age, more severe cough, combining with chronic sinusitis, cough easily triggered by exercise, and abdominal muscle pain due to cough were risk factors for UI in adult female patients.

Previous studies have proven that older age was an important risk factor for UI [13, 17, 18, 25–29], which was consistent with our observation. It might be resulted in the decline of urethral sphincter tension as the increase of age [30]. Univariate analysis showed daytime CSS, night-time CSS, and cough VAS scores in patients with UI were significantly higher than that in patients without UI. However, when the above three values were enrolled in multivariate logistic regression analysis, it was found that only the cough VAS score could be an independent risk factor. Hence, the cough VAS score might reflect the cough severity generally, and might be possible to correct for the daytime and nighttime CSS.

There are two possible reasons for “cough easily triggered by exercise” (defined as intentional exercise beyond daily activities in this study) being a risk factor for UI [31–36]. Firstly, muscle tightness could increase abdominal pressure during daily exercise, especially during moderate-to-high intensity exercise. Meanwhile, cough triggered by exercise could continuously increase abdominal pressure then increase the risk of UI. Secondly, mild-to-moderate physical exercise can train the function of pelvic floor muscles. A normal pelvic floor muscle tension is the key to offset the excessive increase in abdominal pressure after exercise, which is helpful to prevent UI. If the cough is easily induced by exercise, patients will be resistant to do exercises, which makes it difficult to train the pelvic floor function adequately. Additionally, with the increased abdominal pressure due to prolonged coughing, the risk of UI will be certainly increased.

This study also showed that “chronic sinusitis” was an independent risk factor for UI. That chronic sinusitis is often characterized by nasal congestion, runny nose, dysosmia and facial pain, is common in patients who have been diagnosed with allergic rhinitis, asthma, chronic obstructive pulmonary disease, and other respiratory diseases [37]. But up to now, there is no direct evidence that chronic sinusitis was a risk factor of UI. Bekele, et al. reported that the incidence of UI in pregnant women with respiratory diseases such as asthma, allergic rhinitis and rhinosinusitis is higher than those without respiratory diseases [38]. A few studies have shown that severe nasal symptoms such as sneezing and runny nose in women could induce UI [19, 39, 40]. However, in our study, we found that there was no significant difference in the incidence of sneezing and nasal congestion between the patients with UI and patients without UI, we speculated that this is because of the high prevalence of nasal symptoms in Chinese population and the serious air pollution in China, yet there is still lacking of epidemiological researches in this area [41]. We also compared the prevalence of UACS between the patients with UI and patients without UI and found that there was no significant difference in it (4.8% vs. 5.6%, *p* = 0.651). A large-scale epidemiological survey of chronic sinusitis covering 7 cities in China shows that age is a risk factor for chronic sinusitis [37]. Therefore, a large proportion of elderly patients in UI group might increase the incidence of chronic sinusitis, which may also be the reason why chronic sinusitis was a risk factor for UI in this study. However, "chronic sinusitis" was not corrected by "age" in the multivariate analysis. Xie, et al. also found that "rhinitis" was a risk factor for UI in females, but there was still no sufficient evidence to explain the result [13]. Thus, whether there was any overlapping pathogenesis between chronic sinusitis and UI needed further study.

The study also found that abdominal muscle pain due to cough was a risk factor for UI in female patients with chronic cough. The median VAS score of patients with abdominal muscle pain due to cough was significantly higher than that in those without this complication (60 vs. 50, *p* = 0.002), which indicated that patients who had abdominal muscle pain often presented more severe cough symptom, However, "abdominal muscle pain due to cough" was not corrected by "cough severity (VAS)" and remained as an independent risk factor in multivariate analysis. We wondered if it was attributed to the weakness and the lack of tension of the abdominal wall muscles and the pelvic floor muscles in these patients so that persistent cough was more likely to cause more severe damage to the muscles, ultimately lead to UI. Nevertheless, the specific mechanisms and interconnections are needed to be confirmed by further studies.

We analyzed the relationship between the distribution of cause of chronic cough and the occurrence of UI, and found that neither single nor compound causes could significantly increase the risk of UI. Chronic coughers with any cause showed similar susceptible to developing UI, and if the risk factors mentioned above were considered together, the occurrence of UI would increase. With univariate analysis, it has been found that the patients with UI were more sensitive to environmental allergens such as dust, oil fume, cigarette smoke, cold air, but all of these factors were wiped off the list after using multivariate analysis. Thus, no significant correlation between allergen-induced cough and UI was found in our study.

In this study, the reasons why BMI failed to enter the multivariate model might be related to the subjects, the numerical distribution, and, most importantly, the large number of missing data. Yet, previous studies have shown that high BMI was a strong predictor of the presence of UI in female [13, 17, 18, 25–29]. Noblett et al. considered that overweight and obesity could cause the pelvic floor in a chronic state of increased pressure, thereby increase the risk of UI, by illustrating the relationship between BMI and intra-abdominal and intravesical pressure [42]. Even if BMI was not included in the multivariate analysis at the end, we can still believe that overweight and obesity were very important for UI developing by combining the results of the univariate analysis.

There were also some limitations in this study. First of all, some potential factors which could affect female pelvic floor dysfunction including surgical history (e.g. delivery and gynecological surgery), and the number of deliveries were not recorded, it might make an influence on the comprehensiveness of the result of the study. Additionally, the severity and duration of UI were not evaluated, resulting in nonsignificant effect of the risk factors. It has been reported that stress UI was more commonly presented [43, 44], thus it would be worth analyzing whether there were differences in the risk factors of different types of UI, and future study could focus on this question. Thirdly, the details of the treatment history were not recorded, which made it difficult to analyze the impact of treatment on UI. Meanwhile, this study was a single-center retrospective study, and some missing variables could not be verified, the relationship of the demographic, clinical factors and UI might not be comprehensive and accurate. However, considering the large sample size in this study, our findings could provide a helpful and referenced guiding for the management of chronic coughers. Lastly, as our sample consists of patients visiting a specialist cough clinic, it might not be representative to chronic cough patients in the community. But in our study, most of the patients were presented with the common causes of chronic cough, and the proportion of unexplained cough or chronic refractory cough was similar as previous reports [7]. Furthermore, we found that the CSS and cough VAS were similar among different causes in this study. The etiological distribution did not play an predominent role in UI. This study still provided an important implication in clinical, but further study aimed at patients from the community should be conducted to confirm it in the future.

## Conclusion

In conclusion, older age, more severe cough, chronic sinusitis, cough easily triggered by exercise and abdominal muscle pain due to cough are the risk factors for urinary incontinence in female patients with chronic cough. We should pay more attention to the female patients who showed a possibility of developing urinary incontinence in clinical practice. It’s necessary for female patients to strengthen their pelvic floor muscle function in daily life to reduce the incidence of urinary incontinence. A prospective epidemiological investigation with larger sample on urinary incontinence was needed to conduct in the future, while the physiological structure and functional indicators should be taken into consideration as important risk factors.

## Supplementary Information

**Additional file 1:** Case Report Form for adult female patients with chronic cough.
**Additional file 2:** Multivariate logistic regression analysis on risk factors of UI in female patients with chroniccough (after variables reduction).

## Data Availability

All data generated or analyzed during this study are included in this published article.

## Abbreviations

UI: Urinary incontinence
BMI: Body mass index
CVA: Cough variant asthma
EB: Eosinophilic bronchitis
GERC: Gastroesophageal reflux cough
UACS: Upper airway cough syndrome
AC: Atopic cough
UC: Unexplained chronic cough
CRC: Chronic refractory cough
VAS: Visual analogue scale
CSS: Cough symptom score
