# Supplementary material for: The risk factors for urinary incontinence in female adults with chronic cough
Source: BMC Pulm Med. 2022 Jul 18;22:276. doi: 10.1186/s12890-022-02069-w (PMC9295435; doi:10.1186/s12890-022-02069-w)
Supplement: Supplementary file 1 — Additional file 1: Case Report Form for adult female patients with chronic cough. Additional file 2: Multivariate logistic regression analysis on risk factors of UI in female patients with chroniccough (after variables reduction). [file 12890_2022_2069_MOESM1_ESM.pdf]

Additional file of *The risk factors for urinary incontinence in female adults with chronic cough*

Additional file 1: Case Report Form

Name of Researcher：___________ Date:_______________

Basic information

1. Name： ___________
2. Birth Date： _________________
3. Gender：□Male □Female
4. Phone： _______________

Medical History

5. Duration：____year and____month（____months）
6. Timing of cough：□Daytime □Before sleep □Night（After sleep）
   □Moring
7. Seasonal：
   □No □Yes（○Spring ○Summer ○Autumn ○Winter） □Not applicable （Duration＜2 years）
8. Cough feature：
   □Dry cough
   □Productive cough Sputum features:________
9. Daytime CSS：_________ Nighttime CSS：_________

The criteria of Cough symptom score

Daytime Cough symptom score	Nighttime Cough symptom score
0=no cough during the day	0=no cough during the night
1=cough for one short period	1=cough on waking only
2=cough for more than two short periods	2=wake once or early due to cough
3=frequent coughing, which did not interfere with usual daytime	3=frequent waking due to coughs activities
4=frequent coughing, which did interfere with usual daytime activities	4=frequent coughs most of the night
5=distressing coughs most of the day	5=distressing coughs preventing any sleep

10. Cough VAS：0 1 2 3 4 5 6 7 8 9 10

11. Feature of cough frequency：□A single cough □Continuous cough

12. Abdominal muscle pain due to cough：

    □No

    □Yes

13. Cough triggered by meals：

    □No

    □Yes （○Before meal ○After meal ○During meal）

14. Cough triggers

    □No

    □Yes（○Dust ○Cooking fume ○Cold air ○Common cold ○Supine position ○Cigarette smoke ○Exercise ○Talking ○Alcohol ○Others___________）

15. Concomitant symptoms：□No □Yes

15.1 Pharyngeal symptoms：□Itchy throat □Itching below the throat □Pharyngeal foreign body sensation □Throat clearing

15.2 Nasal symptoms：□Sneezing □Rhinocnesmus □Nasal congestion □Postnasal dripping

15.3 Symptoms of lower airway： □Chest tightness □Shortness of breath

15.4 Gastrointestinal symptoms： □Acid regurgitation □Nausea □Belching

□Heartburn □Upper abdominal pain

15.5 Others： ________________

16. Comorbidities： □Allergic rhinitis □Chronic sinusitis □Gastrointestinal disorders □Hypertension

17. History of medication： □Inhaled corticosteroids □Oral corticosteroids □Antitussive drugs

Additional file 2:

Based on previous reports and our preliminary results by excluding BMI, 10 variables including "age, duration of cough, cough VAS, Daytime CSS, Nighttime CSS, a single cough, combining with chronic sinusitis, concomitant with sneeze, cough triggered by exercise, abdominal muscle pain due to cough" were enrolled in the multivariate analysis eventually.

Multivariate logistic regression analysis on risk factors of UI in female patients with chronic cough (after variables reduction).

	Multivariate analysis		
	OR	95% CI	*P value*
Age(years)			＜0.001
Ⅰ 18-30	1		
Ⅱ 31-40	1.900	1.011-3.568	0.046
Ⅲ 41-50	3.252	1.697-6.233	＜0.001
Ⅳ 51-60	3.689	1.934-7.037	＜0.001
Ⅴ ＞60	4.735	2.382-9.412	＜0.001
Cough VAS	1.019	1.010-1.028	＜0.001
Combining with chronic sinusitis	1.827	1.051-3.176	0.033
Cough triggered by exercise	1.843	1.161-2.927	0.010
Abdominal muscle pain due to cough	3.076	2.002-4.725	＜0.001

VAS, Visual analogue scale; CI, Confidence interval.
